# Enhancement of muscle and locomotor performance by a series compliance: A mechanistic simulation study

**DOI:** 10.1371/journal.pone.0191828

**Published:** 2018-01-25

**Authors:** Jason W. Robertson, Colin N. Struthers, Douglas A. Syme

**Affiliations:** Department of Biological Sciences, University of Calgary, Calgary, Alberta, Canada; Brown University, UNITED STATES

## Abstract

The objective was to better understand how a series compliance alters contraction kinetics and power output of muscle to enhance the work done on a load. A mathematical model was created in which a gravitational point load was connected via a linear spring to a muscle (based on the contractile properties of the *sartorius* of leopard frogs, *Rana pipiens*). The model explored the effects of load mass, tendon compliance, and delay between onset of contraction and release of the load (catch) on lift height and power output as measures of performance. Series compliance resulted in increased lift height over a relatively narrow range of compliances, and the effect was quite modest without an imposed catch mechanism unless the load was unrealistically small. Peak power of the muscle-tendon complex could be augmented up to four times that produced with a muscle alone, however, lift height was not predicted by peak power. Rather, lift height was improved as a result of the compliance synchronizing the time courses of muscle force and shortening velocity, in particular by stabilizing shortening velocity such that muscle power was sustained rather than rising and immediately falling. With a catch mechanism, enhanced performance resulted largely from energy storage in the compliance during the period of catch, rather than increased time for muscle activation before movement commenced. However, series compliance introduced a trade-off between work done before versus after release of the catch. Thus, the ability of tendons to enhance locomotor performance (i.e. increase the work done by muscle) appears dependent not only on their established role in storing energy and increasing power, but also on their ability to modulate the kinetics of muscle contraction such that power is sustained over more of the contraction, and maximizing the balance of work done before versus after release of a catch.

## Introduction

Animals that employ single, ballistic body movements rely on contraction of skeletal muscle to load their tendons, aponeuroses, and other series compliances that subsequently move the body. Some invertebrates that employ catapult jumping lock their limbs in place with a mechanical catch mechanism while their muscles contract and impart energy to load tendons or other elastic structures [1–6]. Likewise, animals that employ ballistic tongue and head movements for prey capture use muscle to load compliant tissues and then release the energy through a brief, explosive recoil of the compliance [7–9]. Many other animals that jump, particularly vertebrates, do not appear to possess a mechanical catch mechanism that holds the body in place as muscle loads the compliance [10,11], but instead rely on so-called inertial or dynamic catch mechanisms [10,12,13], where the inertia of body mass, the poor mechanical advantage of extensor muscles crossing joints early in the jump, and perhaps co-activation of antagonist muscles, act to impede movement of the body and serve as a functional catch mechanism. Along with these catch mechanisms, it is proposed that tendons and other series compliant structures uncouple movement of the muscle from the load, allowing the energy from muscle contraction to be stored in the compliance and released at a later time and at a much higher rate than the muscle might otherwise achieve on its own, resulting in high power and high velocities of movement to enhance locomotor performance [2,6,10–29].

Modeling studies have revealed a number of insights into the role played by tendons in enhancing movement [12,16,17,22,30–35]. They have highlighted the role of tendons in uncoupling movement of the muscle from the load [10,12,22,35–37], allowing the possibility of increased power from the muscle-tendon complex (MTC) compared to what a muscle alone can produce. The magnitude of the increase in power afforded by tendons is mathematically unlimited given appropriate design, approximately 40% with a gravitational load alone [30,33] and 20% with a fluid load and appropriate mechanical advantage of joints [32]. Very high power output is observed during the remarkable jumps of some frogs [12,18,28] and some invertebrates [3,6,11], and during head movement and tongue projection in lizards, frogs and fish [7–9], and in many cases high power and achievement of high peak velocities appears to be the objective of MTC design. However, high power output *per se* will not necessarily result in enhanced performance for many types of movement, specifically when performance is dependent on the amount of work done on a load. For example, high power but only for a brief period might result in the same amount of work done compared with lower but sustained power [28]. Ultimately, muscle is the sole source of energy during a single, ballistic movement; tendons may increase peak power output but they cannot contribute energy (i.e., work) to the system. Thus, if the ability to produce mechanical work is a key objective in enhancing performance of a movement, the tendon’s influence must ultimately be to increase work done by the muscle on the load [23]. It is in this context that we frame our study.

Here again, modeling studies have been informative. The ability of tendons to store and later release energy, where large amounts of work can be done by the muscle via the tendon’s impact on contraction kinetics, simultaneous with continued power production by the muscle as this stored energy is released, has the potential to increase the total work done by muscle during a cycle of limb movement [32,33]. Further, very precisely timed relationships must exist between various facets of muscle contraction (e.g. onset/duration of activation, time-course of force and power output), loading of tendons with strain energy, and movement of the load, for enhancements in performance to be realized [16,32,33,35]. To increase work done, the tendons must interact with the load and muscle in a manner that enhances the ability of the muscle to do work, by altering the dynamics of muscle contraction [29]. It is this role of tendons that we further explore in the present study. Our objectives were to assess: 1) how tendon compliance impacts work and power from muscle and tendons and how this relates to locomotor performance (load lifting), and 2) specifically how tendon compliance interacts with load mass and mechanical catch delay to influence muscle contraction kinetics in a manner that results in increased work done and imparted to the load. This was accomplished by modeling interactions between a muscle, tendon and load, using contractile characteristics based on measurements from muscle, to understand what circumstances of muscle contraction are altered by tendons to ultimately enhance the work done on the load. It is revealed that work is enhanced through an effect of the tendon on muscle contraction that sustains muscle shortening velocity, although not force, at a relatively constant level through much of the contraction, resulting in power output being sustained over more of the contraction than occurs with a tendon that is excessively or inadequately compliant. Mechanical catch mechanisms result in high muscle power (and work output) during the period of catch, with more work done during the period of catch than is done after release of the catch, and more work than can be done by a muscle that is connected directly to the load.

## Materials and methods

### Simulation framework

An iterative, computational modeling approach was employed to simulate a muscle lifting a load vertically and linearly via a tendon. This framework was adopted as an intuitive means to reflect work done by the muscle on a load (i.e. lift height is a proxy for muscle work done on the load). The simulations were not intended to model jumping per se, but rather to reflect how a series compliance might affect any ballistic locomotor performance via an influence on muscle contraction. The muscle started in a resting (i.e., not contracting) state with the load in a stationary position. The inertia and weight of the load, and in some simulations a mechanical catch, resisted upward movement of the load. In the model, series compliance resides entirely within the tendon. The modeling program was developed in LabVIEW (v6.1, 8.0.1, and 8.2.1, National Instruments, Austin, TX, USA). The model and equations used are described in detail in the S1 Appendix. In brief, extension of the tendon is based on its physical dimensions, its material properties, and the force exerted on it. Movement of the load is based on its weight and the upward force exerted by the muscle via the tendon. The model could also simulate a mechanical catch, which would prevent the load from moving until a set time had elapsed following muscle activation (catch delay). The force produced by the muscle is based on its physical dimensions (muscle mass was fixed at 10 g, initial muscle length at 10 cm), maximal isometric stress (250 kN m^-2^), time after the onset of activation (force-time relationship), muscle length (active and passive force-length relationships), and shortening velocity (force-velocity relationship), properties that were measured from the sartorius muscle of leopard frogs (*Rana pipiens*). The force produced by the muscle is exerted on the tendon and load, which in turn causes extension of the tendon and acceleration of the load. The velocity of muscle shortening is a function of both movement of the load and extension of the tendon. Thus, the variable *V* in the Hill [38] equation (S1 Appendix) can be substituted with velocity of the load and tendon elongation. As such, the equation describing muscle force as a function of velocity, and the equation describing tendon force as a function of elongation, both contain tendon elongation as a common variable. This allows the equations to be solved for a unique solution that will simultaneously satisfy the effects of tendon and load movement on muscle force, and the effects of muscle force on tendon elongation and load movement, during each iteration of the simulation. This solution can then be used to determine force, tendon elongation, velocity of the muscle and load, and numerous other parameters for each iteration of the model.

These calculations were made over a series of discrete 100 μs time intervals, which was sufficiently short to approximate a continuous system (see S1 Appendix). A given simulation ended at the point where the muscle could no longer generate force, at which point ballistic equations were used to determine the maximal height the load would eventually attain based on its current velocity and height. This process was repeated across a range of parameters describing the tendon, muscle, and load, which could be systematically varied over a user-defined range to determine their effect on performance. These parameters included the initial length of the muscle as a percentage of its optimal length *L*_*0*_, the length of the tendon, the tendon cross-sectional area, the Young’s elastic modulus of the tendon (*E*), the mass of the load, and the time delay before release of the catch. These parameters could be varied in pairs over a user-defined range in 100 linear increments for muscle length and catch delay, or 100 geometric increments for all other parameters, producing 10,000 simulations using the different combinations of the two parameters. The maximal lift height attained in each simulation, as a proxy of muscle work done on the load and hence the level of performance of a task, was then plotted against the associated parameters, producing three-dimensional surfaces showing how lift height was affected by the parameters. Note, in some of the figures only a portion of the full surface that was simulated is shown to emphasize the region of greatest interest. To assess the effects of compliance on muscle contraction, and in turn how they influence work done and lift height, the values of the various outcome variables over the entire time course of selected simulations are also presented, rather than just the final outcome of the simulation.

### Tendon

The tendon was modeled as a Hookean spring (S1 Appendix) [12]. During simulations, the effect of tendon compliance was tested over several orders of magnitude, ranging from highly compliant, where muscle contraction would simply extend the tendon and not move the load, to functionally rigid, akin to the muscle being attached directly to the load. This was accomplished by varying either one or two parameters that defined the tendon, including the Young’s modulus (0.001 to 5 GPa), tendon length (0.1 to 10 cm), or tendon cross-sectional area (0.001 to 1 cm^2^); it was not necessary to vary all three parameters, as altering any one was equally effective in changing the tendon compliance, and combinations of two of the three parameters similarly had equivalent effects (see Results). In most series’ of simulations, either tendon area or Young’s modulus was varied over a wide range of values, so the selection of a particular initial length, area, or modulus of the tendon did not mask results or limit the scope of the investigation. For most simulations, the initial tendon length was set at 2 cm, being 20% of the resting muscle length (10 cm), selected to represent the broad range of relative tendon lengths observed in animals, but not to replicate any particular animal or MTC. In some simulations, the tendon cross-sectional area was fixed at 0.95 mm^2^ (1% of muscle cross-sectional area, assuming a muscle density of 1.05 g cm^-3^), consistent with observations in many mammals [39,40]. The Young’s modulus in vertebrate tendons is typically about 1.5 GPa [17,41,42]. The amount of energy absorbed by the tendon during elongation, and released during recoil, was calculated as the sum of the products of change in tendon length and average tendon force over each iteration interval of the simulation.

### Load

The load was modeled as a gravitational point mass. It was initially at rest and supported by the surface on which it rested, and could not fall below this resting position. During simulations, the mass of the load was either fixed at 20% of the maximal isometric muscle force, where the muscle would produce 85% of maximal power, or systematically varied over the range 0.001% to 100% of maximal force to assess the effects of load mass on muscle work, power, and lift performance.

### Muscle

Contractile properties of the *sartorius* muscle of leopard frogs (*Rana pipiens* complex, *n* = 5) were recorded from living tissue *in vitro* for use in the simulations. All procedures for animal handling were approved by the University of Calgary Animal Care Committee, following Canadian Council on Animal Care guidelines (protocol BI2002-062). Frogs were purchased from a commercial supplier and housed in tanks with a flow-through water supply at room temperature (18–20°C), open water for swimming, and a platform raised above the water. Frogs were fed mealworms supplemented with nutrient powder daily, and were provided daily inspections by facility staff and routine veterinary inspections. The frogs were killed by rapid pithing of the brain and spinal cord. Anesthetic was not used to avoid the depressive effects of anesthetics on muscle contraction. The *sartorius* was isolated in ice-chilled physiological saline (concentrations in mM: NaCl, 115; KCl, 3; CaCl_2_, 2; NaHCO_3_, 20; NaH_2_PO_4_, 2; glucose, 5; pH 7.8 at 20°C, bubbled with 96% O_2_ and 4% CO_2_), leaving the origin attached to a small piece of the ilium and the insertion attached to its tendon. Surgical silk was tied to the ilium and the free tendon, with the knots placed as close to the muscle fibres as possible to minimize stray compliance. The muscle was then placed in an experimental chamber filled with physiological saline at 20°C. The free tendon was tied to the arm of a servomotor (model 305 B-LR-LC, Aurora Scientific Inc., Aurora, ON, Canada), which controlled muscle length. The segment of ilium at the opposite end of the muscle was tied to a stainless steel pin attached to a force transducer (Entran ELG-V-500G, Entran Sensors and Electronics, NJ, USA).

Platinum plate electrodes were placed on each side of the muscle for electrical stimulation. The plates were connected to a wet-cell battery that was gated by a custom-built, Darlington-transistor follower circuit that was in turn gated by a computer-controlled stimulator (Grass SD-9, Astro-Med Inc., West Warwick, RI, USA). The muscle was stimulated with a 1 ms pulse at increasing voltage levels until force was maximal, and the stimulus voltage was then adjusted to 150% of that value to ensure complete recruitment of the muscle. For tetanic stimulation the pulse frequency was 100 Hz. The servomotor and stimulator were controlled by a computer with a 12-bit data acquisition and control card (PCI-MIO-16E4, National Instruments, Austin, TX) and custom software (LabVIEW v6.1, National Instruments). Muscle length, force, and the stimulus were recorded on the same system at a sampling rate of 5.0 kHz.

Simulated muscle force was calculated based on three parameters [43] measured from the muscles: 1) the force-length relationship, both active and passive; 2) the force-velocity relationship; and 3) the force-time (i.e. activation) relationship which describes the kinetics of force rise following activation. The isometric force-length relationship was measured by recording maximal, developed, isometric tetanic force over a range of muscle lengths in 1 mm increments, with a 4 minute rest between each contraction. Resting (passive) force was also recorded at each length. Optimal muscle length (*L*_*0*_) was defined as the length where maximal isometric force was attained. For each muscle, force was normalized to maximal force and muscle length to *L*_*0*_. The data from all muscles were then pooled and a normalized, and the active force-length relationship was fit with a third-order polynomial regression, although in simulations active muscle force was constrained to be nonnegative (i.e. the simulation was stopped if muscle length attained a value where active force would be less than zero). Likewise, a pooled, normalized, passive force-length relationship was fit with a third-order polynomial regression; in the simulations, passive force was constrained to be zero at lengths less than *L*_*0*_. These normalized force-length equations were then used to calculate scaling factors in the simulations, characterizing the active and passive force-producing capacity of the muscle as a function of muscle length.

The force-velocity relationship was measured as described previously [44], with passive tension being subtracted from force in each measurement to yield only developed force. Force values were normalized to maximal isometric force, and velocity values to muscle lengths per second. The pooled force-velocity data was then fit to a Hill equation [38]. The normalized force-velocity equation was solved for muscle force and served as a scaling factor in the simulations, characterizing the force-producing capacity of the muscle as a function of shortening velocity.

When a muscle is activated, force rises from rest to maximal with a specific time course. This force-time (activation) relationship was determined by holding muscle length at *L*_*0*_ and applying a tetanic stimulation until maximal force was attained. These force records were normalized to maximal force and then averaged to generate a single array of data at a resolution of 5 kHz. This array was then used as a scaling factor in the simulations, characterizing the force-producing capacity of the muscle as a function of time after onset of activation. For simulated time points between discrete data points in the array, scaling values were calculated via linear interpolation.

## Results

### Physiological properties of the muscle

The physiological values recorded from the frog muscle preparations are reported as mean ± standard error of the mean. Maximal, isometric, tetanic force averaged 288 ± 7.99 kN m^-2^. The parameters *a* and *b* from the normalized Hill force-velocity equation, calculated from combined data from all five preparations, were 0.52 *P*_*0*_ and 4.17 muscle lengths s^-1^, respectively. *V*_*max*_ averaged 7.60 ± 0.092 muscle lengths s^-1^, and maximal isotonic power averaged 296 ± 16.9 W kg^-1^. Eqs (1) and (2) below describe the polynomial regressions relating normalized active force (*P*_*a*_; *r*^2^ = 0.95) and passive force (*P*_*p*_; *r*^2^ = 0.91) to percentage of optimal muscle length (%*L*_*0*_). Constants are shown rounded to three significant digits in the equations below, however, 15 digits (i.e., double precision) were used in the computer simulations.

$$P_{a}\;=\;-5.88+0.151\;{\%L}_{0}-9.54\;X\;10^{-4}\;{\%L}_{0}^{2}+1.37\;X\;10^{-6}\;{\%L}_{0}^{3}$$ (1)

$$P_{p}\;=\;-3.72+0.125\;{\%L}_{0}-1.38\;X\;10^{-3}\;{\%L}_{0}^{2}+5.07\;X\;10^{-6}\;{\%L}_{0}^{3}$$ (2)

The data used to describe the force-length and force-time (activation) relationships are shown in S1 Fig.

### Effects of tendon compliance

**Fig 1:**
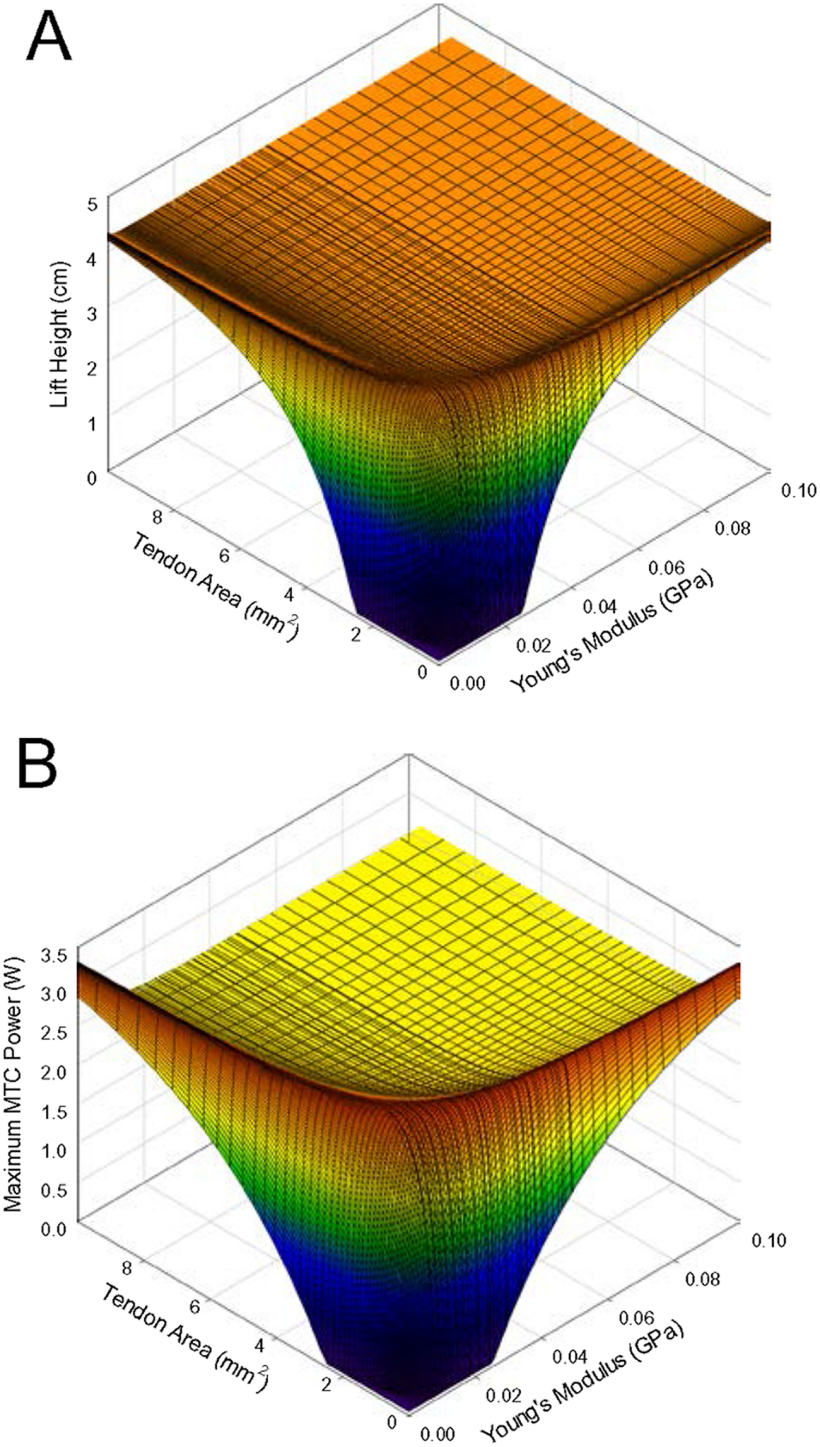
Lift height (A) and maximal MTC power (B) versus tendon area and Young’s modulus. Initial tendon length 2 cm, load mass 485 g, and no catch delay.

The effect of tendon compliance on lift height was tested by examining various combinations of tendon Young’s modulus and cross-sectional area, with load mass fixed at 485 g (20% of maximal isometric force) and no catch mechanism other than load inertia. The greatest improvement in lift height, compared to that produced with a functionally rigid tendon (i.e., one with a very high modulus or area), was a modest 4% (Fig 1A), and resulted from any combination of tendon modulus and area yielding a product of 1.50×10^−4^ GPa cm^2^ or a ratio of tendon modulus to length of 0.79 GPa m^-1^. Thus, varying any one of tendon modulus, length, or area, would suffice to assess the effects of tendon compliance on lift height. The effect of tendon compliance on maximal MTC power output (Fig 1B) revealed a similar pattern to that for lift height, such that the combinations of area and Young’s modulus that maximized power output were similar to those that maximized lift height. The Pearson product moment correlation coefficient between lift height and maximal MTC power was 0.96 (*p* < 0.001) across the entire surface. However, the increase in maximal power, over that produced with a rigid tendon, was 36%, substantially greater than the 4% relative increase in lift height.

### Effects of load mass

**Fig 2:**
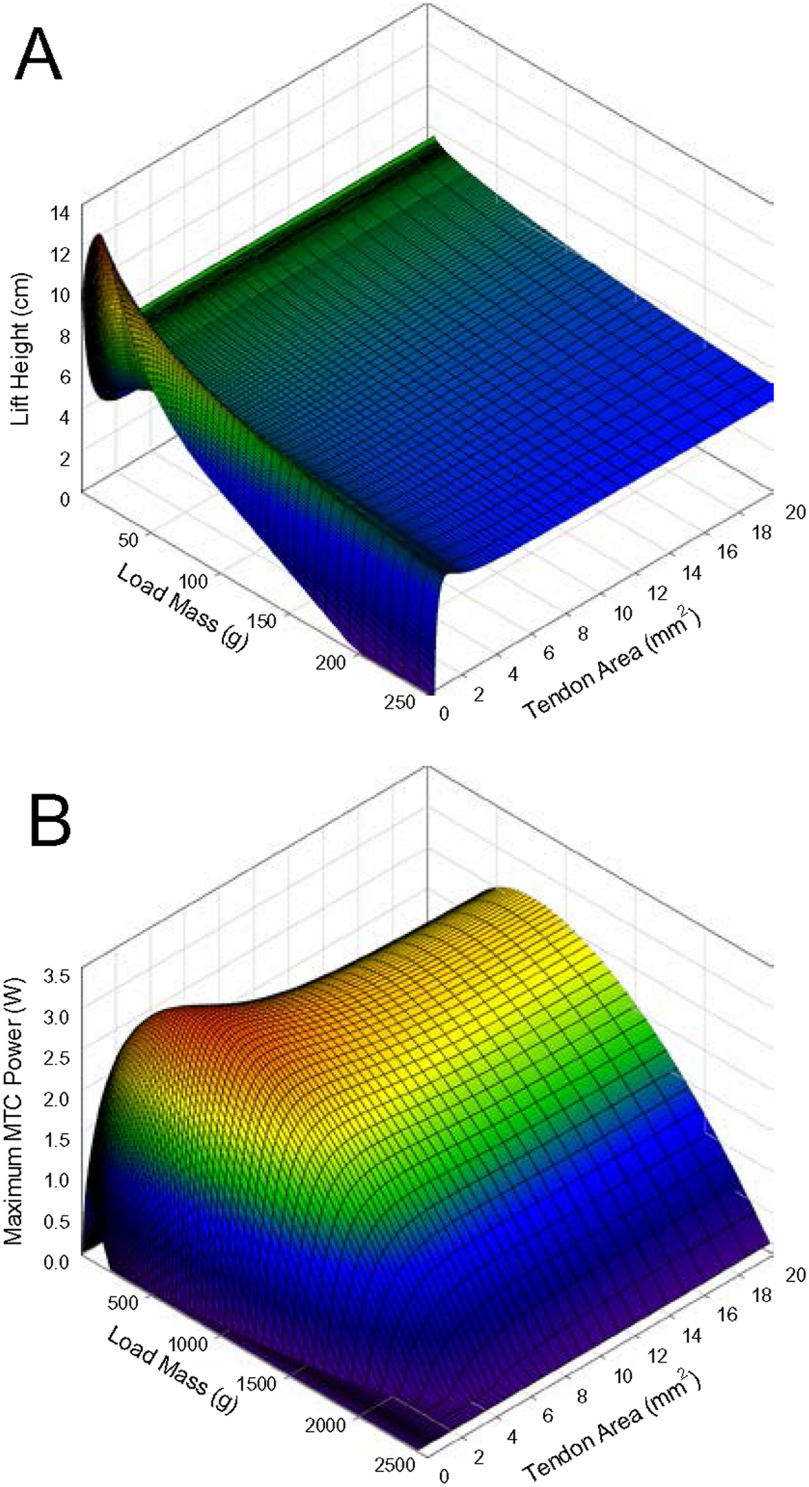
Lift height (A) and maximal MTC power (B) versus load mass and tendon area. Tendon Young’s modulus 0.01 GPa, initial tendon length 2 cm, and no catch delay.

The results above only reveal the lift performance with a 485 g mass, which is unlikely to be the maximum possible for the specific muscle under study. By varying both the load mass and tendon compliance, assessment of the maximal increase in performance is possible (Fig 2A). The increase in height attained with a tendon of appropriate compliance, relative to that with a stiff tendon, varied with load mass. The maximal lift height attained under the range of parameters studied was 13.03 cm, achieved with a mass of 12.14 g (about 0.5% of maximal force), which represents a 93% increase over the lift height of 6.76 cm achieved with a rigid tendon and the same mass. This gain in lift height due to tendon compliance decreased progressively with load masses above or below 12.14 g. Thus, load lifting performance was maximized by a specific combination of compliance and load mass, not simply reduced mass, and the increase in performance was substantial when inertia alone acted as a catch mechanism, but only with a very light load.

When a tendon of appropriate compliance was employed, maximal MTC power also increased above that generated with a functionally rigid tendon (Fig 2B), and the relative improvement was also load-dependent. A maximal increase in power output of 29% (3.23 W with a suitably compliant tendon vs. 2.50 W with a functionally rigid tendon) was recorded with a load mass of about 500 g; as with lift height, the improvement in power output declined with an increase or decrease in load mass beyond that point, approaching zero at relatively heavy and light loads. However, the patterns of change in maximal power and lift height with respect to load mass and compliance were visibly different (compare Fig 2A and 2B); note that the range of load masses displayed in Fig 2B has been extended relative to Fig 2A to provide a clearer depiction of the relationship between load and power. The Pearson product moment correlation coefficient between lift height and maximal MTC power was -0.187 (*p* < 0.001) across the entire surface, confirming the lack of a direct relationship.

### Effects of a catch delay

**Fig 3:**
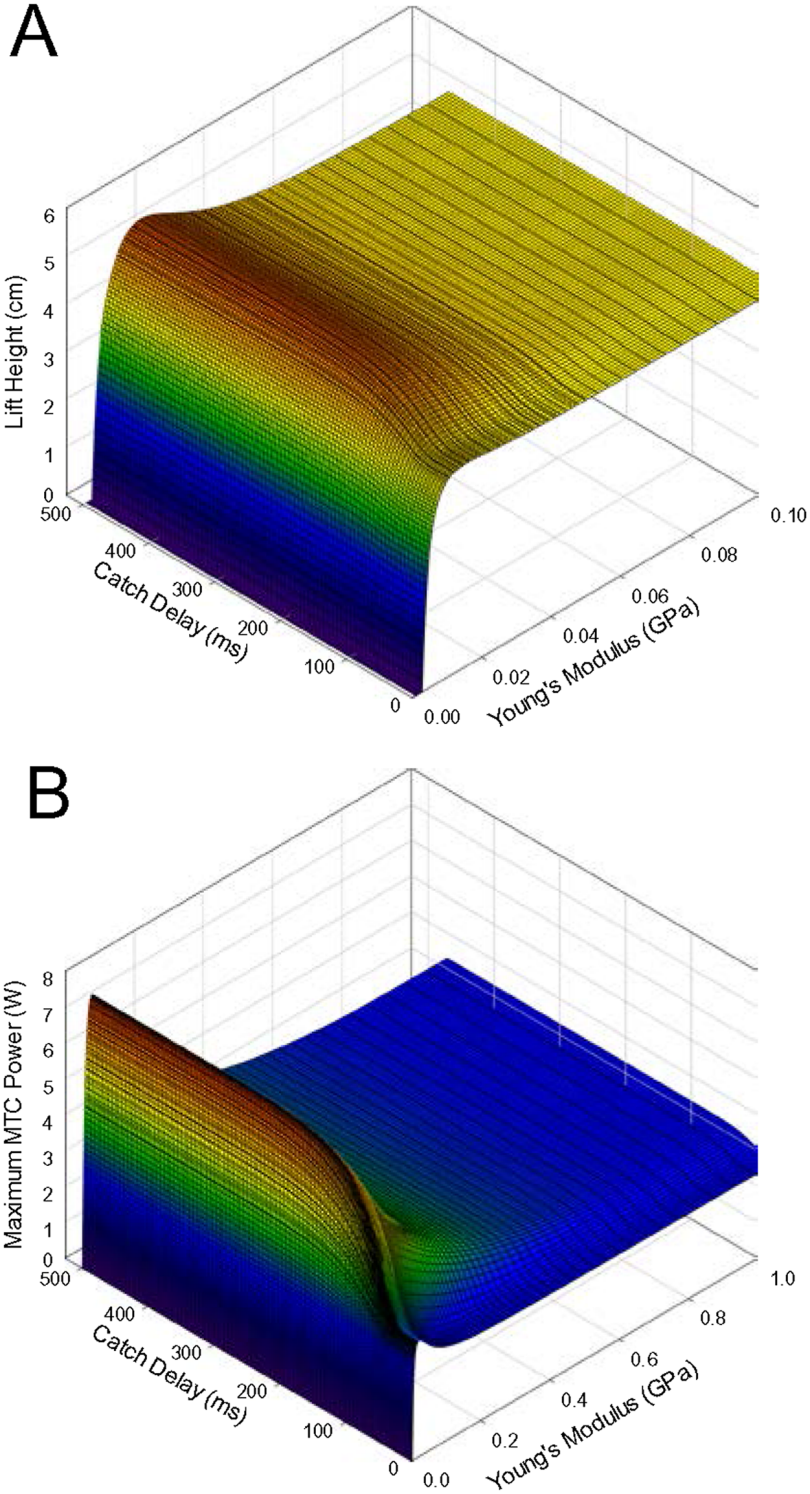
Lift height (A) and maximal MTC power (B) versus catch delay and tendon Young’s modulus. Initial tendon length 2 cm, and load mass 485 g. Panel B has an extended rage of tendon Young’s modulus compared with panel A to illustrate the more gradual fall in power than lift height with reduced compliance.

The effect of a catch mechanism, that delayed load movement for a set period of time following the onset of muscle contraction, was tested with the load mass fixed at 20% of maximal isometric force and over a range of tendon compliances (Fig 3A). A catch delay between 0 and about 25 ms produced the same results as having no catch mechanism at all (cf., Fig 1A), as the muscle had not yet developed enough force to lift the load during this period. Increasing catch delay beyond 25 ms yielded a progressive increase in lift height, with the majority of the improvement occurring over the first 200 ms. With a relatively stiff tendon (*E* = 0.10 GPa) lift height increased by just 1% with increasing catch delay, suggesting that allowing the muscle more time to become activated before release of the load had little effect on lift performance. However, with the ideally compliant tendon (E = 0.0167 GPa) there was a 24% improvement in lift height with increasing catch delay, and overall a 29% greater lift height compared with no delay and a rigid tendon, suggesting that the majority of improvement accrued through events that occurred during the catch period in the presence of a compliant tendon.

Maximal MTC power output showed a pattern of change similar to that for lift height (Fig 3B). Maximal power with a relatively rigid tendon increased from 2.37 W with no catch delay to 2.67 W with a 500 ms delay, an increase of only 13%. However, when a tendon with a Young’s modulus of *E* = 0.0306 GPa was employed (that resulting in maximal power output), power increased by 135%, from 3.17 W with no delay to 7.45 W with a 500 ms delay. Thus, the total power increase from conditions with no delay and a rigid tendon to a long delay and appropriately compliant tendon was 214%. Note that the relative magnitude of the power increase (214%) was far greater than that of lift height (29%), and that the ideal tendon compliance for power generation (E = 0.0306 GPa) was about double that for lift height (E = 0.0167 GPa). The Pearson product moment correlation coefficient between lift height and maximal MTC power was 0.822 (*p* < 0.001) across the entire surface.

### Effects of catch and load mass

**Fig 4:**
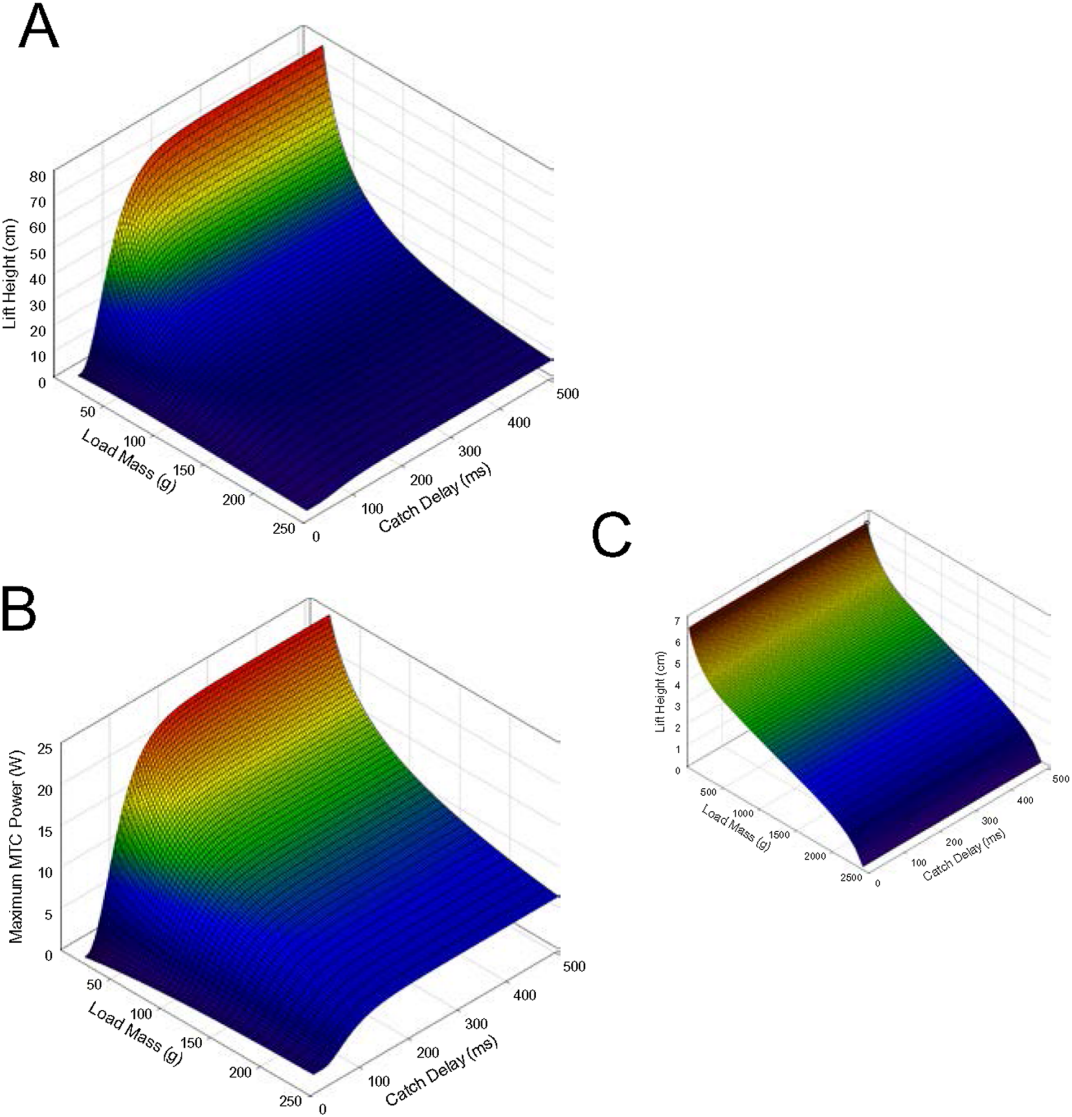
Lift height (A, C) and maximal MTC power (B) versus load mass and catch delay. Panels A and B show results with a compliant tendon (*E* = 0.01 GPa), while C shows lift height with a functionally rigid tendon (*E* = 5 GPa) and an expanded range of load masses. Initial tendon length was 2 cm.

A final set of simulations was undertaken to assess the effects of varying both catch delay and load mass on lift height. In these simulations, tendon modulus was fixed at 0.01 GPa, near that at which lift height was maximal in the previous test. Lift height increased with reduced load mass, but most notably with longer catch delays (Fig 4A). With relatively light loads (10–50 g), lift height increased with catch delay by over an order of magnitude. Running the simulation with a functionally rigid tendon (*E* = 5 GPa) resulted in a pronounced difference in both the magnitude of lift height attained and the pattern of response to these variables (Fig 4C): in this case, lift height was an inverse and near linear function of load mass, with little effect of catch delay, and the maximal lift height was less than one-tenth that attained with a compliant tendon.

When employing a compliant tendon, maximal MTC power increased with both catch duration and reduced load mass, except at very short catch delays where power tended to decrease with reduced load mass (Fig 4B). The relative increase in power was similar to the relative increase in lift height with altered loads and delays. The Pearson product moment correlation coefficient between lift height and maximal MTC power was 0.959 (*p* < 0.001) across the entire surface with a compliant tendon, but only 0.791 (*p* < 0.001) with a rigid tendon.

### Mechanisms relating compliance, power, work, and lift height

**Fig 5:**
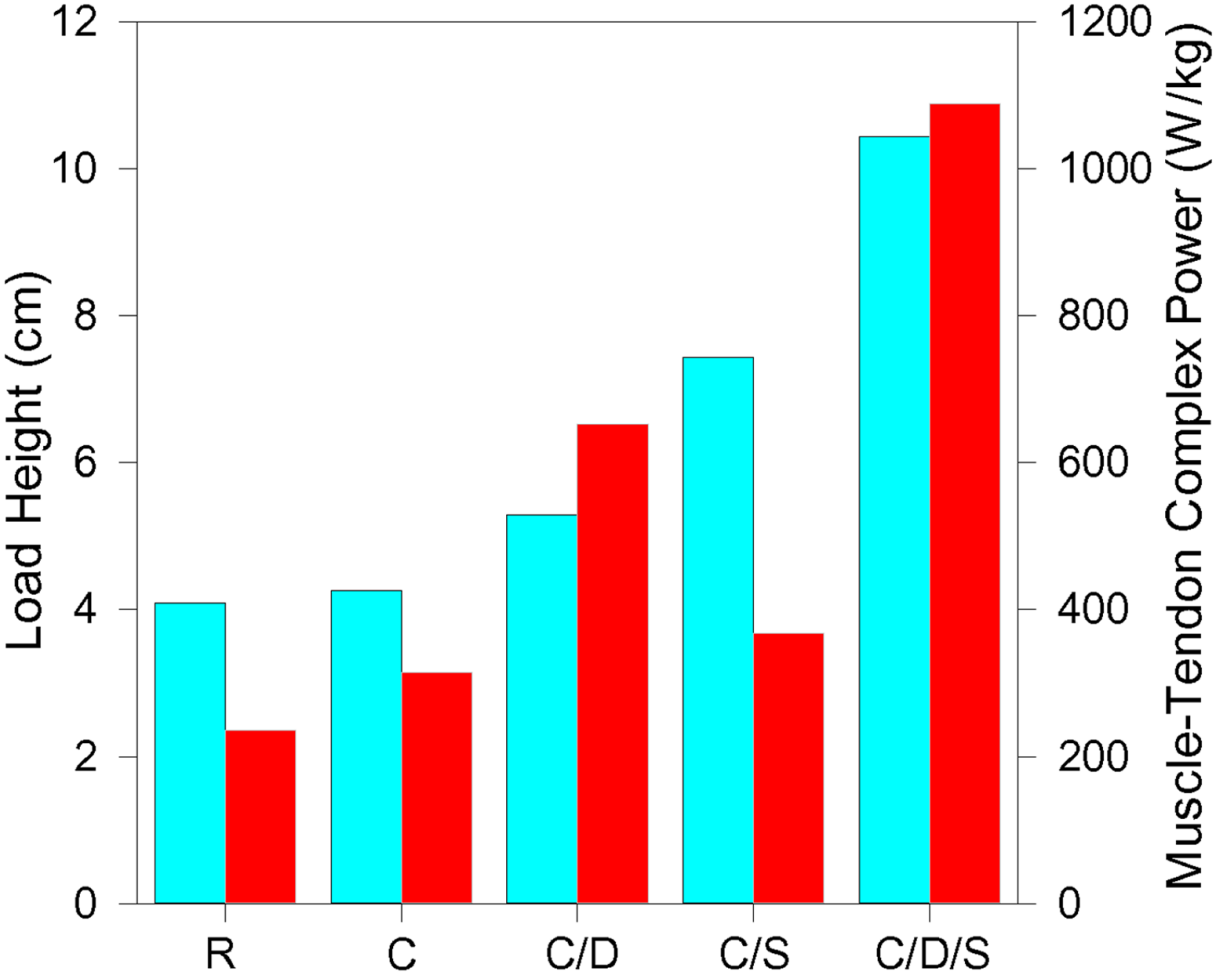
Maximal load height attained (cyan) and associated maximal MTC power (red). Data are from simulations with a 485 g load mass and a tendon with a compliance that maximized lift height. *R*–muscle connected to load with a functionally rigid tendon (*E* = 5 GPa). *C*–muscle connected to load with a compliant tendon (*E* = 0.0159 GPa). *C/D*–muscle connected to load with a compliant tendon (*E* = 0.0167 GPa) and 500 ms catch delay. *C/S*–muscle connected to load with a compliant tendon (*E* = 0.00505 GPa) and initial muscle length 120% *L*_*0*_. *C/D/S*–muscle connected to load with a compliant tendon (*E* = 0.00909 GPa), 500 ms catch delay, and initial muscle length 120% *L*_*0*_. Initial tendon length was 2 cm for all simulations.

To assess how lift height, as an proxy for the work done by muscle and hence the performance of a task, is related to peak power output, the relationship between lift height and maximal MTC power was assessed via correlation using all the results produced within each series of simulations, as reported in the preceding sections, and also by selecting only the highest lift from within each series of simulations for comparison with its associated MTC power (Fig 5). This latter comparison included an additional series of simulations in which the initial muscle length was set to 120% of *L*_*0*_ so that contraction started on the descending limb of the force-length relationship, as has been observed in jumping frogs [45]. As with the correlation analyses reported above, there was not a consistent relationship between maximal MTC power output and maximal lifting performance; changes in some parameters produced similar improvements in both lift height and power, while others produced opposing or unrelated changes.

**Fig 6:**
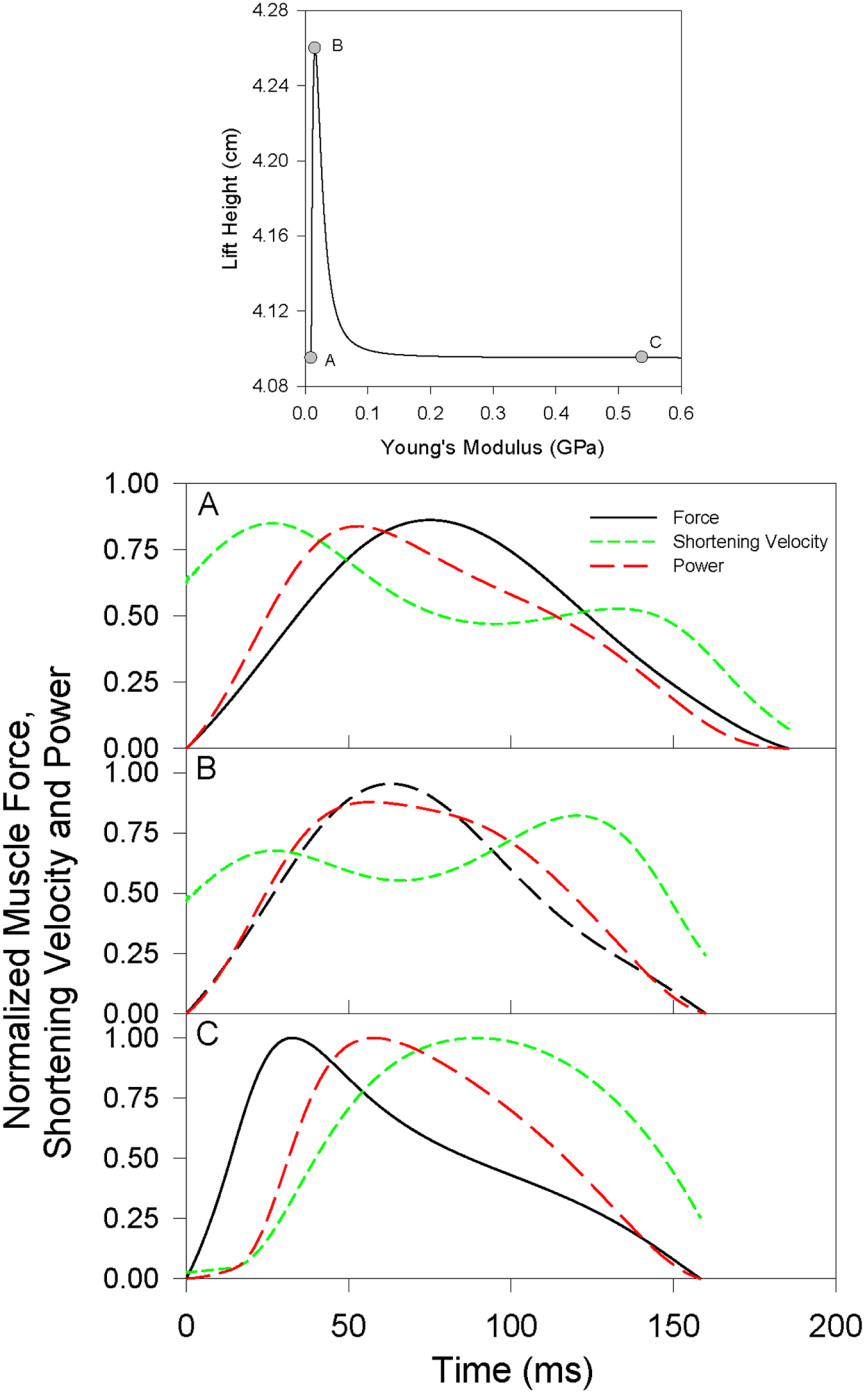
Relative muscle force (solid, black), muscle shortening velocity (short dash, green), and power (long dash, red) with tendons of different compliance. Tendon A was excessively compliant, tendon B was ideally compliant for maximizing work, and tendon C was insufficiently compliant (i.e. rigid). All data are normalized to the maximal values obtained with a functionally rigid tendon (Panel C). Upper pane shows the load lift heights associated with Tendons A, B and C. Tendon A *E* = 0.00968 GPa; Tendon B *E* = 0.0159 GPa; Tendon C *E* = 0.538 GPa. Initial tendon length 2 cm; load mass 485 g, no catch delay.

While peak power only represents power at a moment in time, the integral of power over time yields muscle work production. Broadly speaking, the work done on a load to accelerate it upwards will be directly proportional to the maximal height it attains. Therefore, the effects of tendon compliance on the ability of muscle to do work will directly impact lift performance, and are assessed here. Three points along the lift height profile of Fig 1A, using the same parameters of load (485 g) and catch delay (none), were selected for more detailed analysis of the effects of tendon compliance on muscle power and its components, muscle force and muscle shortening velocity: one point where the stiffness of the tendon resulted in maximal lift height (Fig 6B), and two other points where lower but equal lift heights were attained given a relatively high compliance tendon (Fig 6A) or a low compliance tendon (Fig 6C). The highly-compliant Tendon A tended to shift the primary peak of muscle shortening velocity early in the contraction, where the muscle readily stretched the compliant tendon, and the peak in force later in the contraction, where the muscle then accelerated the load through the stretched tendon. As a result, power rose to a peak between the peaks of velocity and force but then immediately began to fall during the remainder of the contraction. The low-compliance Tendon C (functionally rigid) did the opposite, shifting the peak in force earlier in the contraction where the muscle accelerated the load from a stationary state through the rigid tendon, and shifted the peak in velocity of muscle shortening later in the contraction where the muscle shortened rapidly along with the moving load. As a result, power again rose to a peak between the peaks of force and shortening velocity, then immediately began to fall. With the ideally-compliant Tendon B, the velocity of muscle shortening remained more consistent throughout the contraction, still exhibiting a double peak but with the two peaks being similar in amplitude, and muscle force was likewise maximal near the middle of the contraction rather than early or late. As such, relatively high force production coincided with a moderate and relatively steady shortening velocity over a sustained period, allowing high power to be sustained through more of the contraction than during the simulations with relatively high or low compliance. As a result, the muscle with Tendon B did more work (0.203 J) than the muscle with Tendons A and C (0.195 J).

**Fig 7:**
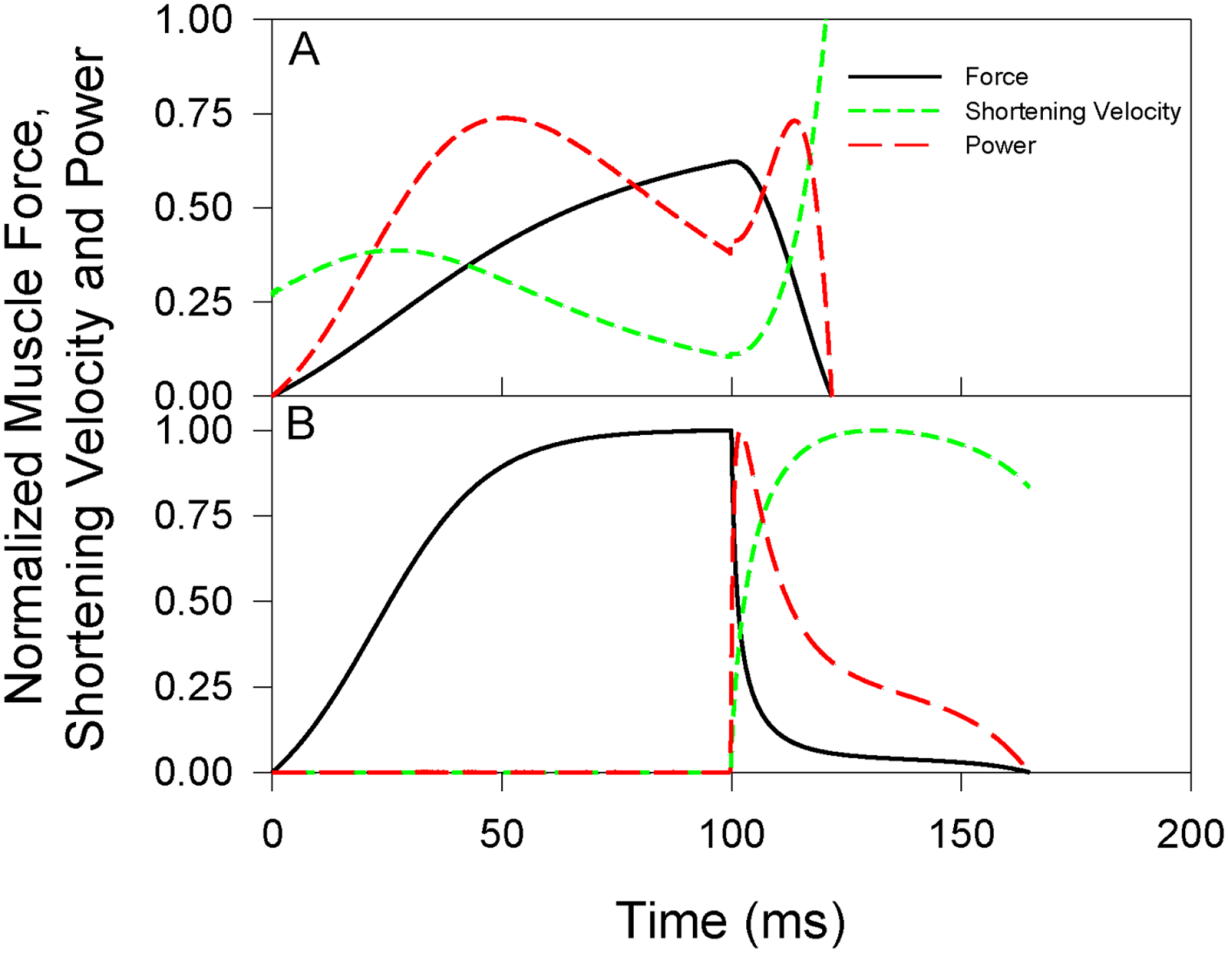
Relative muscle force (solid, black), muscle shortening velocity (short dash, green), and power (long dash, red) with a 100ms catch delay. Panel A had a compliant tendon (*E* = 0.0159 GPa) and panel B had a functionally rigid tendon (*E* = 5 GPa). Load mass 100 g, and initial tendon length 2 cm. Data are normalized to the maximal values obtained with a rigid tendon (Panel B).

Finally, the effect of a 100 ms catch delay, on the order of the delay between the onset of muscle activation and liftoff in jumping frogs [10,13,45,46], on the mechanisms of work and power production with compliant and rigid tendons was examined (Fig 7). In these simulations, work and lift height with a suitably compliant tendon were 2.9 times greater than those with a functionally rigid tendon. With the compliant tendon (Fig 7A), the muscle stretched the tendon and produced substantial power during the 100 ms catch period. Following release of the catch, the energy stored in the tendon rapidly accelerated the load to a peak velocity of 169 cm s^-1^, 222% of the muscle’s maximal velocity of shortening (*V*_*max*_). Muscle shortening velocity also rose swiftly after release, and thus muscle force and power quickly fell to zero. Hence, the majority of work done by the muscle was imparted to the tendon before the catch was released, and relatively little work was done by the muscle after the release. With the non-compliant tendon (Fig 7B), while the muscle generated high levels of force during the catch period, it could not shorten, so power was zero and thus no work was done. Following release of the catch, the muscle shortened and accelerated the load very rapidly. This produced a brief period of high power, with a peak about 25% higher than was produced with the compliant tendon, and with more work done after release of the catch than was done by the muscle associated with the compliant tendon. However, after release of the catch power was quickly inhibited by the high shortening velocity, and thus low force, of the muscle. The work done by the muscle was therefore limited to the brief period of load acceleration after release of the catch. Further, the maximum velocity of the load was limited by the shortening velocity of the muscle; the load could only move as fast as the muscle shortened, peaking at 69 cm s^-1^, or 91% of *V*_*max*_.

## Discussion

### Justification and limitations of the model

Our model simulates a muscle with a fixed origin and an insertion that translates with the tendon and load in a linear fashion. This differs from typical limb geometry where the tendon acts across a joint, and the complex geometry of other systems such as tongue projection where muscle interacts with a compliance to move the load (see Introduction). Roberts and Marsh [12] and Astley and Roberts [13] discuss the potential role of a rotating joint, which produces a time-dependent mechanical advantage, in MTC performance, showing that changing effective mechanical advantage of the joint and inertia can act as catch mechanisms that could enhance movements such as jumping. Such catch mechanisms differ from the abrupt release of the load employed in the present study, which is more akin to the abrupt release of the limb in animals such as jumping fleas and locusts [2,14], and tongue projection [7,8] or head rotation [9]. However, as noted in other modeling studies [32], while the geometry and catch mechanism employed in the simulations may differ in detail from some animals, it is fundamentally similar to what occurs in all animals: catch mechanisms allow energy to be stored and released in elastic structures and uncouple muscle and load movement, allowing insight into how tendons might enhance performance in principle.

The *sartorius* muscle was selected for the model because it is convenient for assessing contractile mechanics in isolated muscles. The *sartorius* is a knee flexor that does not contribute to jumping, and is comprised of approximately 36% amphibian type I, 27% type I/II, and 37% type II fibres, compared with over 80% type I fibres in muscles that power jumping in leopard frogs [47]. This difference will necessarily impact muscle power production: for example, type I fibres produce 382 W kg^-1^, while type II fibres produce 198 W kg^-1^ in African clawed frogs, *Xenopus laevis* [48]. However, the objective of the present study was to describe the effects of tendon compliance on muscle performance, measured as the ability to lift a load, and not a particular muscle or system of jumping. Thus, selection of a specific muscle from a specific species was not of great consequence to interpretation, other than muscles with different complements of fiber types would have different contractile characteristics and so result in different specific combinations of tendon characteristics and load masses that maximize performance.

The specific characteristics of tendons (e.g. Young’s elastic modulus, cross sectional area), catch delay and load mass that resulted in enhanced work and load lifting ability of the muscle should be interpreted with caution when considered in the context of the characteristics found in animals. The load masses and muscle dimensions used in the simulations spanned a wide range of values, with the intent to explore the full range of possibilities for enhancing performance. Further, only two parameters were altered in concert within any given series of simulations, with other parameters left fixed at what would be, for many of the combinations of other parameters, not physiologically relevant values. Thus, in many cases the combinations of characteristics that enhanced performance do not appear to be realistic in terms of what might be found in most animals. This is not to say that only unrealistic conditions appear to result in notable enhancement of work output from muscle, although in some cases this is likely the case (e.g. infinitely small masses result in maximal performance with a catch mechanism). Rather, these results suggests that appropriate combinations can enhance locomotor performance, as has been noted by others, and that this may result via altering the contraction kinetics of muscle such that more work is done, both with and without a dedicated catch mechanism.

Three elements considered to have major impacts on force produced by muscle, force-length, force-time, and force-velocity [43], were included in the model, similar to Roberts and Marsh [12]. As with other modeling studies [12,16,30,32], other elements impacting force production were excluded from the model for simplicity and lack of sufficient information to model them accurately. While this may result in some inaccuracy in estimating work and power output, it likely had little effect on the patterns of response and interpretation thereof. Of note, length-tension properties of the muscle, which have been excluded from some other modeling studies [30,32], were included in the simulation because muscle shortening, and thus length-tension properties, impact the ability of muscle to produce force and do work over the course of such contractions and so were considered important for assessing the effects of compliance on muscle contractile function.

Another potential limitation is that the tendons were modeled as purely elastic structures, whereas tendons are viscoelastic and exhibit hysteresis. Estimates of hysteresis range from 3–20% [41,42], averaging about 7% [17]. This would result in the model slightly overestimating the amount of work transferred to the load and hence absolute lift height, although it is unlikely to have had a notable impact on interactions with muscle contraction and the ability of muscle to do work, which was the major focus of this study. However, some caution should be exercised in attempting to infer quantitative estimates of performance of jumping muscles in a limb using results of this study.

### Mechanistic basis of increased lift performance

When considered in the context of jumping, maximal performance is attained by maximizing the kinetic energy (i.e., velocity) of the animal at takeoff, which is enabled by increasing power output from the MTC [17]. This can occur when muscles are able to impart energy to extend tendons at relatively low velocities of shortening, at which muscle force and thus work is high, followed by tendon recoil releasing that energy quickly, producing both a high power output and a high velocity of movement, effectively circumventing the force-velocity limitations of the muscle. However, as a tendon can only release the energy imparted to it by the muscle, increases in jump height can only be realized if a tendon allows the muscle to do more work than it otherwise could. Such a role has previously been proposed for series compliant structures [12,17,22,23], and may indeed be a general pattern in many animals [23,49]. In humans, this mechanism has been observed in walking [22,50], and subsequently proposed and measured during jumping [12,16,27,51].

Previous modeling studies have examined the role of tendons in altering power generation in several ways. Some have described the role of tendons in uncoupling movement of muscles and the load, allowing muscles to contract at relatively slow velocities where they can impart higher forces and thus do more work [12,22,30,35]. Galantis and Woledge [30] established the notion that tendons store and release energy to increase power and velocity of movement in ballistic movements, and described the extent to which tendons may enhance power output. Other studies have demonstrated that the timing of muscle contraction and tendon extension/recoil are critical in determining if a tendon will enhance or reduce power output from the muscle [16,32,35]. In the present study, we sought further insight into the specific aspects of muscle contraction that are altered by tendons and catch mechanisms in the context of how they allow more work to be done by the muscle. Elements under investigation included the significance of increasing peak power versus the time course of power output for increasing work done, the specific aspects of contraction dynamics that are altered to allow more work to be done by muscle, and the relative contribution of work done by muscle during the period of catch versus after release.

Modeling studies have demonstrated that incorporating a compliant tendon in series with muscle can result in MTC power exceeding that generated by muscle alone (Fig 5) [12,30,33], supporting the notion that tendon compliance could be responsible for the remarkable power output observed in, for example, jumping frogs [18] or chameleon tongue extension [8]. However, peak power does not reliably predict increased performance of a task, and this was confirmed whether analyzed as the correlation between lift height and peak power across all simulations (see Results), or as a comparison of the highest lifts with their associated peak powers from each series of simulations (Fig 5). This is anticipated, as lift height is a function of the work done on the load, which is the integral of power with respect to time, not simply the peak power output. As noted by Roberts et al. [28], while toads produce less power than frogs during jumps, the period over which that power is expressed is longer in toads, making the total difference in work done relatively small. Below we discuss evidence for how series compliance may effect increased muscle work during ballistic movements by altering the contractile dynamics of muscle to extend the period over which high power is sustained during muscle contraction.

A range of series compliances were found to produce a small increase in lift height relative to that with no series compliance (Fig 1), as has previously been observed [11,22]. However, only a very limited subset of possible compliances were effective at increasing lift height, suggesting it is not simply the capacity to store energy or to slow the velocity of muscle shortening that results in increased work. Rather, a specific impact on the kinetics of muscle contraction appears necessary. Tendons with excess (Fig 6A) compliance resulted in an early peak in muscle shortening velocity, associated with a late peak in force, such that power reached a peak but was not sustained beyond the peak. For tendons with inadequate compliance (Fig 6C), the patterns in force and shortening velocity were reversed, but with a similar result of power not being sustained. The tendon that maximized work (Fig 6B) allowed the muscle to interact with the compliance and load such that shortening velocity remained relatively constant through much of the contraction, resulting in power being sustained at high levels for more of the contraction. A similar pattern of shortening velocity is observed during simulations of swimming where power amplification is evident [32], and during high acceleration in terrestrial running [23]. Unlike shortening velocity, however, even in simulations where muscle work was maximized, muscle force was not maintained at relatively high and constant levels through the contraction, rising to a peak and then immediately falling, regardless of tendon compliance (Fig 6), again similar to what is observed in models of high acceleration [23]. Thus, power and work do not appear to be enhanced by an action of tendons allowing sustained muscle force, but more so shortening velocity, although there is a tendency for force to be maximal toward the middle of the contraction with an ideally compliant tendon, rather than early or late in the contraction, which may contribute to increased work from the muscle.

**Fig 8:**
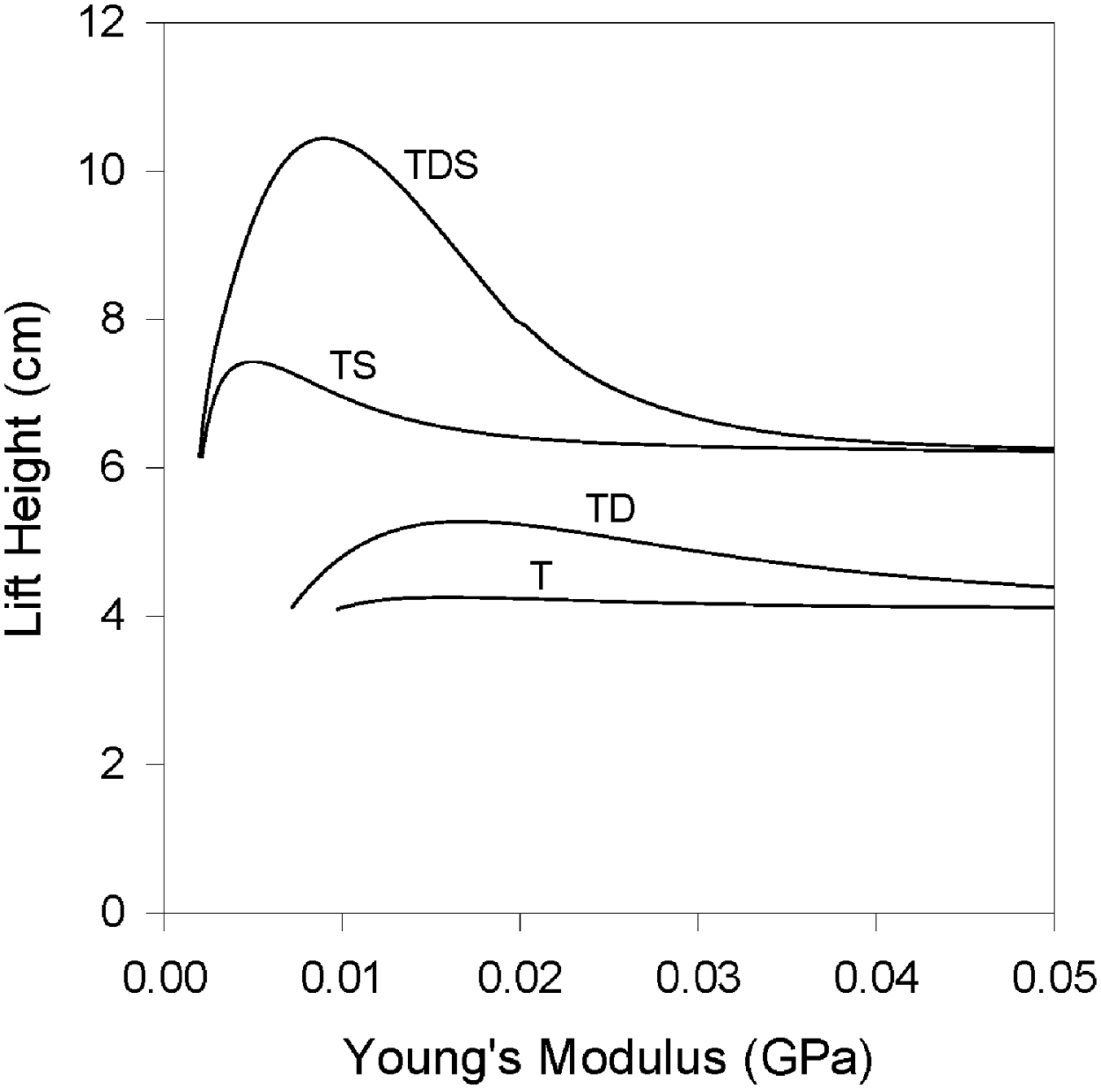
Lift height attained across a range of tendon Young’s moduli with different simulation parameters. *T*–tendon, no catch delay, initial muscle length 100% *L*_*0*_; *TD*–tendon, 500 ms catch delay, initial muscle length 100% *L*_*0*_; *TS*–tendon, no catch delay, initial muscle length stretched to 120% *L*_*o*_; *TDS*–tendon, 500 ms catch delay, initial muscle length stretched to 120% *L*_*0*_. For all simulations, initial tendon length, 2 cm; load mass, 485 g.

Given this proposition, that muscle work and lift height are maximized through a specific interaction between muscle, tendon, and load that promotes sustained power output [23], it follows that the ideal tendon compliance for maximal performance should be different for each different set of parameters (load, catch delay, etc.). This was indeed found to be the case: the maximal lift height attained within each series of simulated parameters was in all cases dependent on the compliance of the tendon, and different parameter sets required different ideal tendon compliances to maximize lift height, even with the same muscle and load (Fig 8). For example, with the same load, stretching the muscle prior to the contraction resulted in a more compliant tendon being ideal, whereas an extended catch delay resulted in a less compliant ideal tendon. Perhaps the increased force that a muscle can produce when working against a stationary load during the catch period allows it to impart more energy to a relatively stiff tendon, while a muscle initially stretched to a longer length gains the capacity to impart more energy to a relatively compliant tendon that will then undergo larger strain. Likewise, the time available for the muscle to load tendons will influence the particular tendon compliance that maximizes performance, where longer times favor less compliant tendons [35]. Further investigation into the transfer of energy between muscle, tendon, and load during these contractions is required to better understand why a particular compliance is of benefit for each combination of load, muscle length, and catch delay.

When a catch mechanism was employed, the muscle attached to the compliant tendon performed about three times the work of the muscle attached to the rigid tendon (Fig 7), despite the muscle with the rigid tendon producing a higher peak power. Most of this work was done before release of the catch; compare the area under the power curves before vs. after 100 ms in Fig 7A. This leads to the possibility that a catch mechanism could benefit muscle work by allowing increased time for muscle activation before work commences on the load. However, when the tendon was relatively stiff (i.e., only the effects of a catch delay and not the effect of compliance is being considered), there was very little increase in the work done by the muscle due to the catch delay (Fig 3A), indicating that increased time for activation per se likely does not contribute notably to improved performance. The catch effect appears more related to the muscle interacting with the series compliance. Further, work done by the muscle is not restricted to only the period during catch [23,32,33]; a substantial amount of work was also done after release of the catch, with both a compliant and rigid tendon. And more work was done after release of the catch when a rigid tendon was employed versus compliant (compare the area under the power curves following 100 ms in Fig 7A and 7B), suggesting that there is potential for a trade-off in enhancing work done before versus after release of the catch with increased tendon compliance. Sawicki et al. [33] note that intermediate loads appear to maximize the sum of work done before and after release of a catch with a series compliance, and that the load itself will influence the merit of using a catch mechanism to enhance ballistic performance. Richards and Sawicki [32] likewise note the importance of the timing of contractile element (i.e., muscle) power and tendon recoil power to overall power output during the leg cycle in models of frog swimming, noting that inappropriate timing will result in power attenuation by series elastic elements rather than power amplification. Further investigation is required to understand how altering the compliance might impact work done before and after release of the catch to maximize total work done by the muscle.

### Summary and conclusions

Previous studies have revealed that the benefits of a series compliance and catch mechanism for locomotor performance stem from the ability of tendons to store energy during the catch period and then to release that energy at a high rate allowing high acceleration and velocity of the load, higher than a muscle itself could attain. Tendons also allow more work to be done by muscle via storage of energy during the catch period, and via allowing muscles to contract in a fashion that allows more work to be done than if they moved the load directly. Here we demonstrate a specific influence that tendons have on muscle contraction kinetics that results in increased work. Tendons of sufficient compliance alter the contraction kinetics such that muscle shortening velocity, but not force, tends to be sustained at relatively consistent levels through much of the contraction, allowing power to be sustained at high levels and hence more work done. Further, peak MTC power was not a reliable predictor of performance; in many cases MTC properties that produced high peak power produced low lift heights, and vice versa. This confirms that time spent generating power, as it relates to work done by the muscle, is more important than the peak power in augmenting lift/jump performance. Catch mechanisms do not appear to yield more work by allowing the muscle more time to become fully activated before the load is released, as the improvement in lift performance with a rigid tendon and long catch delay is very modest over what results with no catch delay. The primary benefit of catch mechanisms appears to be in increasing the ability of muscles to do work before release of the catch, although work done after release of the catch remains significant, and appears to trade-off with the ability to do work before catch release; ideally compliant tendons might balance these two components to maximize total work done.

## Supporting information

S1 Appendix Symbols, derivation, and example validations of the model. (DOCX)

S1 Fig Force-length and force-time (activation) relationships used in modeling muscle contraction. Active (upper panel) and passive (middle panel) muscle force as function of muscle length. Force is expressed relative to maximal active force, and length is relative to the length at which active force was maximal (100%). Regression in red are 3^rd^ order polynomials through the entire data set, and are given in the Results section of the manuscript. The relationship between active, isometric force and time following the onset of muscle stimulation (0 ms) (lower panel); data shown is the average from 5 sartorius muscles of leopard frogs (*Rana pipiens*).
(TIF)

S2 Fig Results of a simulation and the associated force scaling factors. A: Profiles of muscle length, tendon length, load displacement, and muscle force during a simulation of a muscle lifting a load via a compliant tendon. B: Scaling factors used to calculate muscle force relative to its maximal isometric value (*force*) based on the active force-length properties (*length*), force-velocity properties (*velocity*), and force-time properties (*activation*) of the muscle, as derived over the course of the contraction. Initial tendon length 2 cm; tendon Young’s modulus 0.0159 GPa; load mass 485 g.
(TIF)
