# Supplementary material for: Enhancement of muscle and locomotor performance by a series compliance: A mechanistic simulation study
Source: PLoS One. 2018 Jan 25;13(1):e0191828. doi: 10.1371/journal.pone.0191828 (PMC5784993; doi:10.1371/journal.pone.0191828)
Supplement: S1 Appendix — (DOCX) [file pone.0191828.s001.docx]

**S1 Appendix**

## List of symbols

| **Symbol** | **Definition** |
| --- | --- |
| $A$ | Tendon cross-sectional area |
| $a$ | Hill equation constant |
| $\bar{a}$ | Average acceleration of load across previous and current intervals |
| $b$ | Hill equation constant |
| $d_{i}$ | Displacement of the load during the current interval |
| $d_{t}$ | Total displacement of the load as of the current interval |
| $d_{t-1}$ | Total displacement of the load after the previous interval |
| $F$ | Force exerted by the muscle or tendon |
| $\bar{F}$ | Average force exerted by the muscle or tendon across previous and current intervals |
| $F_{max}$ | Maximal, developed, isometric muscle force |
| $F_{net}$ | Net force acting on load |
| $F_{t-1}$ | Force exerted by the muscle or tendon after the previous interval |
| $g$ | Gravitational acceleration |
| $L_{0}$ | Muscle length at which maximal isometric force is developed (optimal) |
| $l_{0}$ | Initial (slack) tendon length |
| ${\Delta l}_{t-1}$ | Total change in tendon length after the previous interval |
| ${\Delta l}_{t}$ | Total change in tendon length as of the current interval |
| $m$ | Mass of load |
| $M$ | Mass of muscle |
| $P$ | Relative muscle force (Hill equation) |
| $P_{0}$ | Maximal relative muscle force (Hill equation) |
| $P_{a}$ | Normalized active force-length scaling factor |
| $P_{p}$ | Normalized passive force-length scaling factor |
| $P_{t}$ | Normalized force-time scaling factor |
| ${\Delta t}_{i}$ | Duration of iteration interval |
| $\bar{v}$ | Average velocity of load across previous and current intervals |
| ${\Delta v}_{i}$ | Change in load velocity over the current interval |
| $v_{t}$ | Velocity of load as of the current interval |
| $v_{t-1}$ | Velocity of load after the previous interval |
| $V$ | Velocity of muscle shortening |
| $V_{max}$ | Maximal velocity of muscle shortening |
| $\rho$ | Muscle density |

## Derivation of model equations

Starting with the Hill (1938) force-velocity equation (A.1), solve for relative force (A.2):

|  | $\left( P+a \right)\left( V+b \right)=\left( P_{0}+a \right)b$ | (A.1) |
| --- | --- | --- |
|  | $P=\frac{\left( P_{0}+a \right)b}{V+b}-a$ | (A.2) |

Muscle shortening velocity is the sum of the upward load velocity and the tendon elongation velocity (A.3). Mean load velocity (A.4) relies on the change in velocity during the current interval (A.5).

|  | $V=\bar{v}+\frac{{\Delta l}_{t}-{\Delta l}_{t-1}}{{\Delta t}_{i}}$ | (A.3) |
| --- | --- | --- |
|  | $\bar{v}=\frac{v_{t}+v_{t-1}}{2}=\frac{v_{t-1}+{\Delta v}_{i}+v_{t-1}}{2}=v_{t-1}+\frac{{\Delta v}_{i}}{2}$ | (A.4) |
|  | ${\Delta v}_{i}=\bar{a}{\Delta t}_{i}$ | (A.5) |

Load acceleration over the interval is a function of the net force acting upon the load, which equals the mean upward force exerted by the tendon less the downward force exerted by gravity (A.6). Define mean force over the interval (A.7), substitute it into (A.6), and solve for acceleration (A.8). Tendon force is a function of its elastic modulus, area, and strain (A.9).

|  | $F_{net}=m\bar{a}=\bar{F}-mg$ | (A.6) |
| --- | --- | --- |
|  | $\bar{F}=\frac{F+F_{t-1}}{2}$ | (A.7) |
|  | $\bar{a}=\frac{\frac{1}{2}\left( F+F_{t-1} \right)-mg}{m}$ | (A.8) |
|  | $F=\frac{{\Delta l}_{t}AE}{l_{0}}$ | (A.9) |

Relative muscle force described by the Hill equation (A.2) was converted to absolute force (A.10) by multiplying *b* by *L_0_* to produce velocity in metres per second, then multiplying by the length and time scaling factors (S1 Fig) and the muscle’s maximal force (A.11) to yield muscle force in Newtons. Passive force was then added. Maximal muscle force is defined by the muscle’s physical and physiological parameters (A.11).

|  | $F=\left[ \frac{\left( P_{0}+a \right)bL_{0}}{V+bL_{0}}-a \right]P_{a}P_{t}F_{max}+P_{p}F_{max}$ | (A.10) |
| --- | --- | --- |
|  | $F_{max}=\frac{M\times250 kN\cdot m^{-2}}{\rho L_{0}}$ | (A.11) |

These equations were then substituted, in order: (A.9) into (A.8), (A.8) into (A.5), (A.5) into (A.4), (A.4) into (A.3), then finally (A.3) into (A.10); this produced equation (A.12), which describes muscle force with Δ*l_t_* as the only unknown. Note that (A.11) was not inserted at any point, as *F_max_* is a constant for any given simulation.

|  | $F=\left[ \frac{\left( P_{0}+a \right)bL_{0}}{\frac{{\Delta l}_{t}-{\Delta l}_{t-1}}{{\Delta t}_{i}}+v_{t-1}+\frac{{\Delta t}_{i}}{2m}\left[ \frac{1}{2}\left( \frac{{\Delta l}_{t}AE}{l_{0}}+F_{t-1} \right)-mg \right]+bL_{0}}-a \right]\times P_{a}P_{t}F_{max}+P_{p}F_{max}$ | (A.12) |
| --- | --- | --- |

The equations describing muscle force (A.12) and tendon force (A.9) were then set equal to each other, as these elements are in series, to solve for the unique value of Δ*l_t_* that would simultaneously satisfy both equations.

To simplify, several elements in the equations were collected and replaced with common factors (A.13)-(A.18) producing equation (A.19).

|  | $X_{1}=\frac{AE}{l_{0}}$ | (A.13) |
| --- | --- | --- |
|  | $X_{2}=\left( P_{0}+a \right)X_{3}$ | (A.14) |
|  | $X_{3}=bL_{0}$ | (A.15) |
|  | $X_{4}=P_{a}P_{t}F_{max}$ | (A.16) |
|  | $X_{5}=v_{t-1}+\frac{F_{t-1}{\Delta t}_{i}}{4m}-\frac{g{\Delta t}_{i}}{2}-\frac{{\Delta l}_{t-1}}{{\Delta t}_{i}}$ | (A.17) |
|  | $X_{6}=\frac{1}{{\Delta t}_{i}}+\frac{X_{1}{\Delta t}_{i}}{4m}$ | (A.18) |
|  | ${\Delta l}_{t}X_{1}=\left( \frac{X_{2}}{{\Delta l}_{t}X_{6}+X_{5}+X_{3}}-a \right)X_{4}+P_{p}F_{max}$ | (A.19) |

Solving for Δ*l_t_* produced a quadratic equation (A.20) that could then be solved using the quadratic formula.

|  | ${\Delta l}_{t}^{2}\left[ X_{1}X_{6} \right]+{\Delta l}_{t}\left[ X_{6}\left( aX_{4}-P_{p}F_{max} \right)+X_{1}\left( X_{5}\boldsymbol{+}X_{3} \right) \right]+\left[ \left( aX_{4}-P_{p}F_{max} \right)\left( X_{5}\boldsymbol{+}X_{3} \right)-X_{2}X_{4} \right]=0$ | (A.20) |
| --- | --- | --- |

Given a physiologically-appropriate value of Δ*l_t_*, force could be calculated using either (A.9) or (A.12). Velocity was found with appropriate variable substitutions, and load displacement could then be determined using velocity:

|  | $d_{i}=\bar{v}{\Delta t}_{i}$ | (A.21) |
| --- | --- | --- |
|  | $d_{t}=d_{t-1}+d_{i}$ | (A.22) |

If load displacement was less than zero, indicating the muscle had not yet generated sufficient force to lift the load, load velocity and displacement were forced to zero and the equations were re-calculated.

## Validating the model

The results of an exemplar lifting simulation (S2 Fig) using a compliant tendon alongside the scaling factors used to calculate muscle force as derived during the simulation shows the simulation behaving qualitatively as anticipated. Muscle force and the activation (i.e. force-time) scaling factor increase rapidly following muscle activation, but force remains less than would be attained under isometric conditions because the muscle shortens as it extends the tendon and displaces the load. The activation scaling factor then plateaus as the muscle attains full activation. Muscle length, and thus the active force-length scaling factor, decreases through the entire simulation, with the rate of decrease depending on extension and recoil of the tendon and displacement of the load. Force then falls as the muscle continues to shorten and the velocity of shortening remains high. The force-velocity scaling factor initially falls as the muscle becomes activated and extends the tendon rapidly, then rises as the extended tendon resists muscle shortening and shortening velocity slows, then falls again as the load accelerates to a high velocity and shortening velocity rises, and finally rises again as the muscle approaches minimal length leading to a slow velocity of shortening. Tendon length increases and decreases in direct proportion to the force exerted on it. The load begins to move when force exceeds load weight, accelerates upward in proportion to the force exerted on it, then continues to rise after muscle contraction ceases due to kinetic energy, before eventually falling back to its resting position.

Four additional simulations were performed to quantitatively assess the program’s ability to model contractions and movements of the tendon and load using parameters that allowed the results of each simulation to be checked against analytical solutions or empirical data. The first test assessed the ability of the model to predict muscle force during an isometric contraction, using an immovable load (i.e., a mass which exceeds the ability of the muscle to lift), and using a functionally rigid tendon (Young’s modulus: *E* = 5000 GPa). The resulting force trajectory (peak force attained 117 ms following activation) and the maximal force attained (23.8 N) matched precisely the values expected given the dimensions specified (10 g mass, 10 cm length, 250 kN m^-2^ specific force) and the time course of force generated during contractions measured previously from living muscle.

The second test assessed the ability of the model to predict the effects of muscle length on force production, and muscle force production on the extension of a tendon. An immovable load was paired with a compliant tendon (*E* = 0.01 GPa). Upon activation, the muscle shortened and the tendon elongated until the muscle’s ability to generate force exactly matched that exerted by the extended tendon, and the contraction became isometric. The resulting muscle length, tendon length, and force at this equilibrium were compared to values calculated directly from the equations describing their force-length relationships and matched precisely.

The third test assessed the effect of force on load movement. The load was set to be light enough for the muscle to lift, and the force applied to it was fixed at a constant value by bypassing the force-time, force-length, and force-velocity equations in the simulation. The expected displacement of the load could then be calculated at any time point using ballistic equations and compared with values reported by the simulation as calculated over many time intervals. It was confirmed that the acceleration of the load was constant, and that after 2000 calculation intervals (200 ms), the two methods agreed within 0.5%, the small difference likely due to rounding errors.

The fourth test assessed energy transfer from the contracting muscle to the tendon and the load, using a load equal to 20% of maximum force and a compliant tendon (*E* = 0.01 GPa). Before the muscle had developed sufficient force to lift the load, work done by the contracting muscle was transferred to elastic strain energy in the tendon. The amount of work done by the muscle was used to calculate the amount of extension that should occur in the tendon, knowing its physical properties; these were confirmed to match. The load commenced moving precisely at the point where the force produced by the muscle was equal to the weight of the load. As the muscle continued to shorten, its ability to produce force began to decline and the tendon began to recoil, transferring energy to the load. When the muscle ceased to produce any force, it was confirmed that the tendon had recoiled to its original resting length, and that the work done by the muscle during the contraction was equal to the sum of the gravitational potential and kinetic energies of the load.

**S1 Fig. Force-length and force-time relationships used in modeling muscle contraction.** Active (upper panel) and passive (middle panel) muscle force as function of muscle length. Force is expressed relative to maximal active force, and length is relative to the length at which active force was maximal (100%). Regression in red are 3^rd^ order polynomials through the entire data set, and are given in the Results section of the manuscript. The relationship between active, isometric force and time following the onset of muscle stimulation (0 ms) (lower panel); data shown is the average from 5 sartorius muscles of leopard frogs (*Rana pipiens*).

**S2 Fig. Results of a simulation and the associated force scaling factors.** A: Profiles of muscle length, tendon length, load displacement, and muscle force during a simulation of a muscle lifting a load via a compliant tendon. B: Scaling factors used to calculate muscle force relative to its maximal isometric value (*force*) based on the active force-length properties (*length*), force-velocity properties (*velocity*), and force-time properties (*activation*) of the muscle, as derived over the course of the contraction. Initial tendon length 2 cm; tendon Young’s modulus 0.0159 GPa; load mass 485 g.
