# Supplementary figures and images for: Enhancement of muscle and locomotor performance by a series compliance: A mechanistic simulation study
Source: PLoS One. 2018 Jan 25;13(1):e0191828. doi: 10.1371/journal.pone.0191828 (PMC5784993; doi:10.1371/journal.pone.0191828)

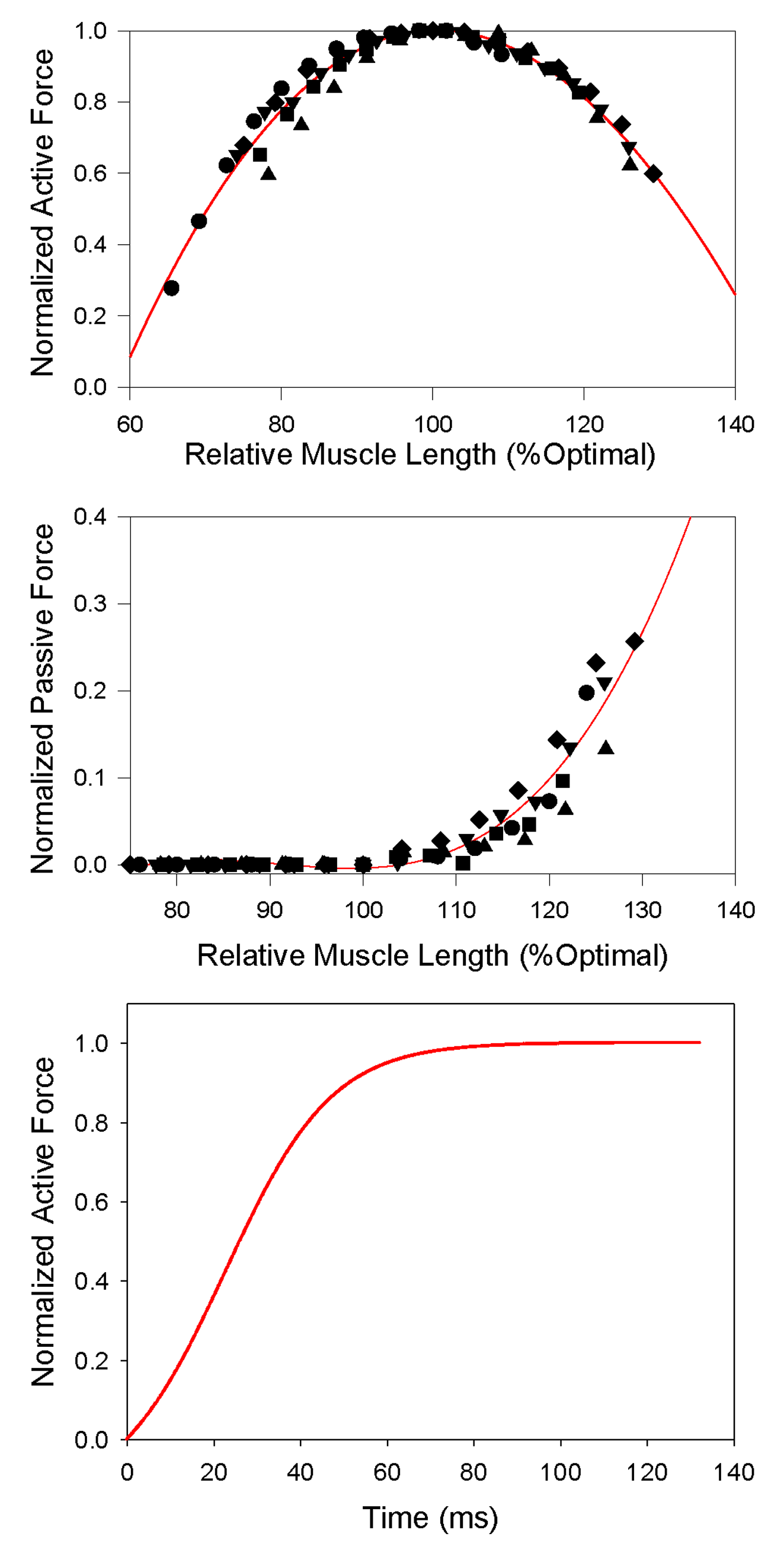

Supplement: S1 Fig — Active (upper panel) and passive (middle panel) muscle force as function of muscle length. Force is expressed relative to maximal active force, and length is relative to the length at which active force was maximal (100%). Regression in red are 3rd order polynomials through the entire data set, and are given in the Results section of the manuscript. The relationship between active, isometric force and time following the onset of muscle stimulation (0 ms) (lower panel); data shown is the average from 5 sartorius muscles of leopard frogs (Rana pipiens). (TIF) [file pone.0191828.s002.tif]

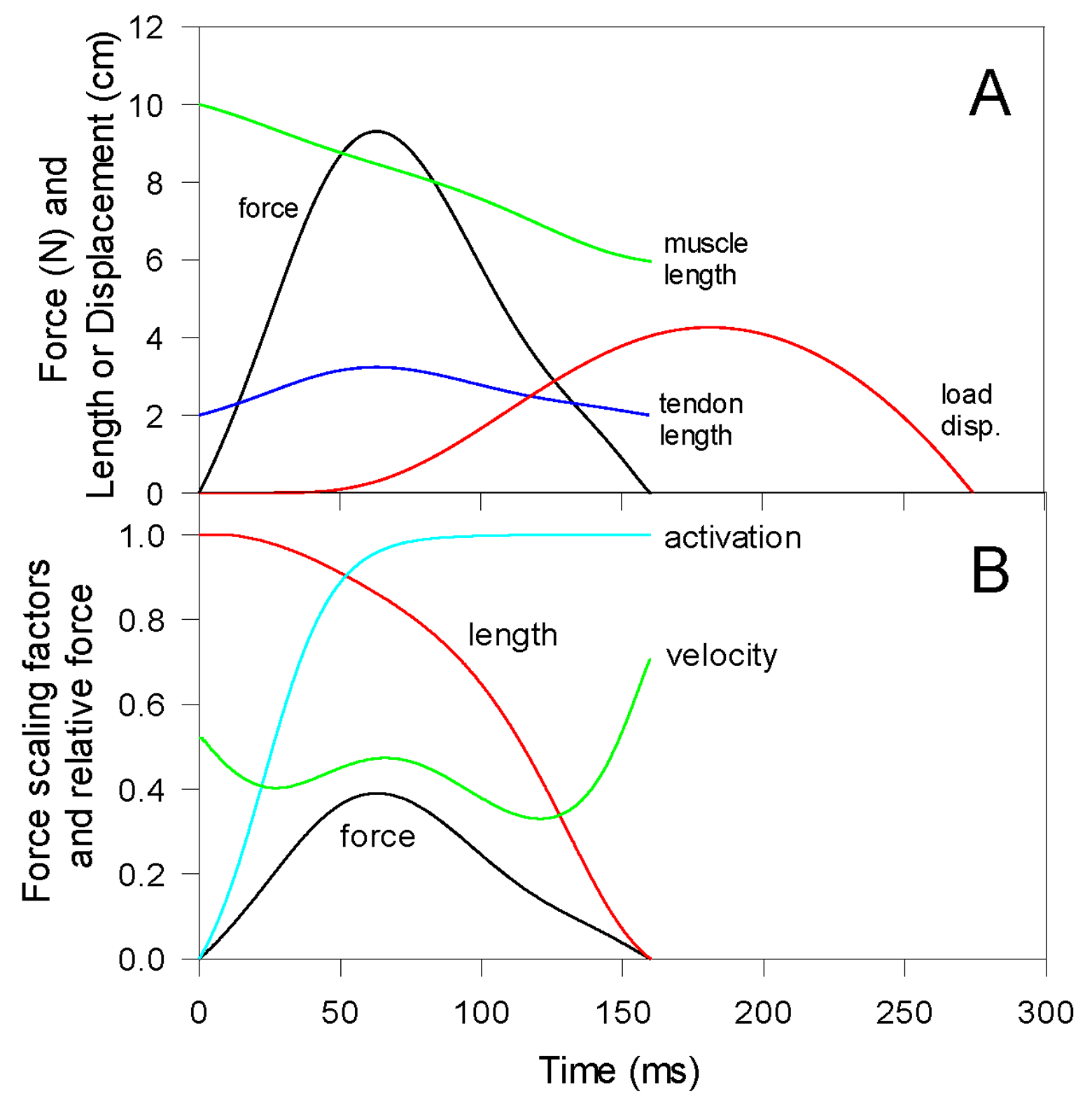

Supplement: S2 Fig — A: Profiles of muscle length, tendon length, load displacement, and muscle force during a simulation of a muscle lifting a load via a compliant tendon. B: Scaling factors used to calculate muscle force relative to its maximal isometric value (force) based on the active force-length properties (length), force-velocity properties (velocity), and force-time properties (activation) of the muscle, as derived over the course of the contraction. Initial tendon length 2 cm; tendon Young’s modulus 0.0159 GPa; load mass 485 g. (TIF) [file pone.0191828.s003.tif]
